# Spectrum of somatic mutations detected by targeted next-generation sequencing and their prognostic significance in adult patients with acute lymphoblastic leukemia

**DOI:** 10.1186/s13045-017-0431-1

**Published:** 2017-02-28

**Authors:** Juan Feng, Yan Li, Yujiao Jia, Qiuyun Fang, Xiaoyuan Gong, Xiaobao Dong, Kun Ru, Qinghua Li, Xingli Zhao, Kaiqi Liu, Min Wang, Zheng Tian, Yannan Jia, Ying Wang, Dong Lin, Hui Wei, Kejing Tang, Yingchang Mi, Jianxiang Wang

**Affiliations:** 1 Institute of Hematology and Blood Disease Hospital, Chinese Academy of Medical Sciences and Peking Union Medical College, Tianjin, 300020 People’s Republic of China; 2 State Key Laboratory of Experimental Hematology, Tianjin, 300020 People’s Republic of China

**Keywords:** Next-generation sequencing, Somatic mutations, Prognostic significance, Acute lymphoblastic leukemia

## Abstract

Target-specific next-generation sequencing technology was used to analyze 112 genes in adult patients with acute lymphoblastic leukemia (ALL). This sequencing mainly focused on the specific mutational hotspots. Among the 121 patients, 93 patients were B-ALL (76.9%), and 28 patients (23.1%) were T-ALL. Of the 121 patients, 110 (90.9%) harbored at least one mutation. The five most frequently mutated genes in T-ALL are *NOTCH1*, *JAK3*, *FBXW7*, *FAT1*, and *NRAS*. In B-ALL, *FAT1*, *SF1*, *CRLF2*, *TET2*, and *PTPN1* have higher incidence of mutations. Gene mutations are different between Ph^+^ALL and Ph^−^ALL patients. B-ALL patients with *PTPN11* mutation and T-ALL patients with *NOTCH1* and/or *FBXW7* mutations showed better survival. But B-ALL with *JAK1/JAK2* mutations showed worse survival. The results suggest that gene mutations exist in adult ALL patients universally, they are related with prognosis.

**Electronic supplementary material:**

The online version of this article (doi:10.1186/s13045-017-0431-1) contains supplementary material, which is available to authorized users.

## To the editor

Acute lymphoblastic leukemia (ALL) represents one of the most common malignant diseases of childhood, accounts for about 15 ~ 25% of acute leukemia in adults [1]. Adult ALL is generally characterized by diverse biological features, evident clinical heterogeneity, and worse prognosis than pediatric ALL [2]. With the development of genetics in ALL, several new subtypes of ALL and a series of prognostic-related molecular markers are put forward [3–5].

In the recent years, with the application of next-generation sequencing (NGS) technology, genomics has been extensively developed in both pediatric and adult ALL patients [6]. Samples and clinical information were collected from 121 adult ALL patients (Additional file 1:Table S1) with informed consent (ethical approval *serial number is* KT2015001-EC-1). These patients were from the Institute of Hematology and Blood Diseases Hospital, Chinese Academy of Medical Sciences. Target regions of 112 genes (Additional file 2: Table S2) were selected on the basis of known or suspected involvement in the pathogenesis of malignant hematologic disorder and were enriched and analyzed using a custom targeted NGS gene panel (Additional file 3). Then, the relationships between the mutations with higher incidence and the prognosis of ALL patients were analyzed (Additional file 4).

**Fig. 1:**
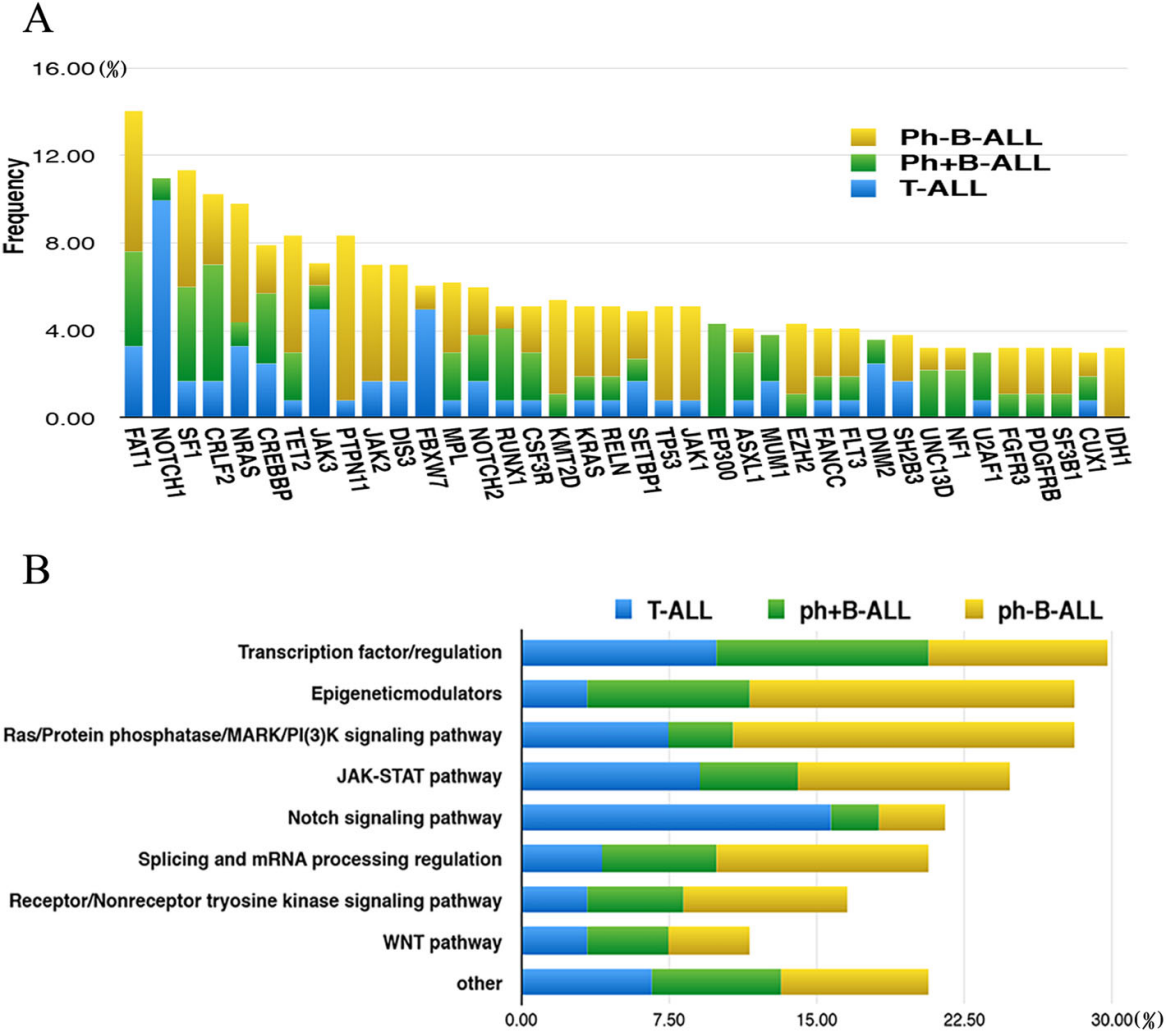
Frequency of gene mutations and related signal pathways in ALL subtypes. **a** Frequency of the top 38 gene mutations in different ALL subtypes, which are shown in indicated colors. **b** Frequency of gene mutations involved in different functional pathways

Of the 121 patients, a total of 110 patients (90.9%) harbored at least one gene mutation with a median of 2 (0–7) mutations per sample. Thirty-nine patients (32.2%) had more than 3 gene mutations (Additional file 5: Figure S1). Sixty genes were considered as possible pathogenic mutations when compared against multiple databases (Fig. 1a. The top 38 mutated genes were listed). The five most frequently mutated genes were *FAT1*, *NOTCH1*, *SF1*, *CRLF2*, and *NRAS* (mutated in >8% of the cases).

In 28 T-ALL cases, the most common mutated gene was *NOTCH1*, with a mutation rate of 39.3% (*n* = 11), then *JAK3*, *FBXW7*, *FAT1*, *NRAS*, *CREBBP*, *DNM2* (mutated in >10% of the cases) (Additional file 6: Figure S2A). In B-ALL, FAT1 was the most accepted mutated gene (10.75%), then *SF1*, *CRLF2*, *TET2*, *PTPN11*, *NRAS*, *CREBBP*, *JAK2*, *DIS3*, *MPL*, and *KML2D* (mutated in >5% of the cases) (Additional file 6: Figure S2B). In Ph^+^ B-ALL, *FAT1*, *CRLF2*, *SF1*, *EP300*, and *CREBBP* genes mutated at higher incidences (Additional file 6: Figure S2C). However, *PTPN11*, *SF1*, *TET2*, *NRAS*, *JAK2*, *DIS3*, and *FAT1* gene mutations occurred popularly in Ph^−^B-ALL (Additional file 6: Figure S2D).

The main signaling pathways involved in this targeted NGS gene panel were transcription factor/regulator, Ras/protein phosphatase/MARK signaling pathway, *JAK-STAT* pathway, splicing and mRNA processing regulation, epigenetic modulators, and so on [7–9]. Frequencies of different signaling pathways involved are listed in Fig. 1b. Genes involved in these signaling pathways are listed in Additional file 7:Table S3.

In full cohort, the median overall survival (OS) was 34.88 (1.25–74.55) months, median relapse-free survival (RFS) was 30.85 (0–73.55) months and 3-year OS and RFS rates were 49%. In the full cohort, patients with *PTPN11* mutation had a better prognosis compared with patients without *PTPN11* mutation (*p* = 0.040, *p* = 0.047), and the patients with *JAK2* mutation (7/117) had a worse prognosis compared with patients without *JAK2* mutation (*p* = 0.031, *p* = 0.018) (Additional file 8: Figure S3). B-ALL patients with *PTPN11* mutation (7/93) had a better OS and RFS compared with those without *PTPN11* mutation (*p* = 0.041, *p* = 0.047) (Additional file 9: Figure S4).

In T-ALL, patients with *NOTCH1* and/or *FBXW7* mutations had a better OS and RFS than patients without these mutations (*p* = 0.035, *p* = 0.048) (Additional file 10: Figure S5).

Multivariate analysis of OS and RFS showed that the prognostic factors included *JAK2* mutations (OS; *p*= 0.045, RFS; *p* = 0.021) in the total adult ALL patients cohort. *JAK1* mutations (OS; *p* = 0.004, RFS; *p* = 0.005) and *JAK2* mutations (OS; *p* = 0.049, RFS; *p* = 0.044) for Ph^−^B-ALL. The data was summarized in Table 1.

**Table 1:** Univariate and multivariate analysis for OS and RFS

	OS		RFS	
	HR^2^	*P* value	HR^2^	*P* value
Univarite analysis (mut/all)				
PTPN11 mut; positive vs negative in full cohort (8/117)	0.043	0.040	0.044	0.047
In B-ALL (7/92)	0.042	0.041	0.042	0.047
In Ph^−^B-ALL (7/54)	0.035	0.030	0.037	0.047
JAK2 Mut; positive vs negative in full cohort (7/117)	3.02	0.031	3.309	0.018
In B-ALL (5/92)	3.033	0.061	3.145	0.051
In Ph^−^B-ALL (5/54)	3.728	0.035	3.780	0.031
JAK1 mut; positive vs negative in Ph^−^B-ALL (4/54)	6.808	0.001	6.562	0.001
NOTCH1 and/or FBXW7 mut; positive vs negative				
In T-ALL (12/25)	0.204	0.035	0.223	0.048
Multivariate analysis				
PTPN11 mut; positive vs negative in full cohort	0.043	0.052	0.044	0.056
In B-ALL	0.042	0.063	0.042	0.056
In Ph^−^B-ALL	0.035	0.178	0.037	0.212
JAK2 mut; positive vs negative in full cohort	3.02	0.045	3.309	0.021
In Ph^−^B-ALL	3.728	0.049	3.780	0.044
JAK1 mut; positive vs negative in Ph^−^B-ALL	6.808	0.004	6.562	0.005
NOTCH1 and/or FBXW7 mut; positive vs negative				
In T-ALL	0.204	0.055	0.223	0.069

In summary, our study suggests that gene mutations exists in adult ALL patients universally, involving a variety of signaling pathways. The frequency and species are varied in different types of ALL. B-ALL patients were accompanied with *PTPN11* mutation for good prognosis, while abnormal *JAK* family often indicates poor prognosis. In T-ALL, mutation of *NOTCH1* and/or *FBXW7* indicates good prognosis.

## Additional files

Additional file 1: Table S1. Demographic data, follow-up time, and ALL subtypes. (DOCX 52 kb)
Additional file 2: Table S2. 112 genes covered by a custom targeted NGS gene panel. (DOCX 94 kb)
Additional file 3: Supplementary: materials and methods. (DOCX 73 kb)
Additional file 4: Supplementary: statistical analysis. (DOCX 52 kb)
Additional file 5: Figure S1. Percentage of ALL patients with different somatic mutations. (TIF 1433 kb)
Additional file 6: Figure S2. Frequency of gene mutations in T-ALL (A), in B-ALL (B), in Ph+B-ALL (C), and in Ph-B-ALL (D). (DOCX 4090 kb)
Additional file 7: Table S3. Signal pathways affected and their frequency. (DOCX 70 kb)
Additional file 8: Figure S3. Probability of RFS or OS for total adult ALL patients with/without *PTPN11* and *JAK2* mutations. (TIF 4888 kb)
Additional file 9: Figure S4. Probability of RFS or OS in adult B-ALL patients with/without *PTPN11* and *JAK2* mutations. (TIF 4909 kb)
Additional file 10: Figure S5. Probability of RFS or OS in adult T-ALL patients with/without NOTCH1 and/or *FBXW7* mutations. (TIF 3370 kb)

## Abbreviations

ALL: Acute lymphoblastic leukemia
CR: Complete remission
NGS: Next-generation sequencing
OS: Overall survival
PCR: Polymerase chain reaction
RFS: Relapse-free survival
SNP: Single nucleotide polymorphisms
WBC: White blood cell
