# Supplementary material for: Spectrum of somatic mutations detected by targeted next-generation sequencing and their prognostic significance in adult patients with acute lymphoblastic leukemia
Source: J Hematol Oncol. 2017 Feb 28;10:61. doi: 10.1186/s13045-017-0431-1 (PMC5331692; doi:10.1186/s13045-017-0431-1)
Supplement: Additional file 1: Table S1. — Demographic data, follow-up time, and ALL subtypes. (DOCX 52 kb) [file 13045_2017_431_MOESM1_ESM.docx]

Additional file 1：Table S1. Demographic data, Follow-up time, ALL subtypes

Parameter Total cohort （n＝121）

Patient numbers

(ratios, ranges or percentages)

Demographic characteristics 121

Males:females (ratio) 79:42 (1.8/1)

Median age (years) 30(14-61)

14-35 71(58.7%)

35-61 50(41.3%)

Cases with follow-up 117

Median follow-up in months 23.95

Median OS in months 16.03

ALL subtypes (WHO, 2008) 121

B-ALL 93(76.9%)

ph+ALL 37(39.8%)

T-ALL 28(23.1%)

Abbreviations: ALL,acute lymphoblast leukemia. OS,overall survival.
