# Supplementary material for: Spectrum of somatic mutations detected by targeted next-generation sequencing and their prognostic significance in adult patients with acute lymphoblastic leukemia
Source: J Hematol Oncol. 2017 Feb 28;10:61. doi: 10.1186/s13045-017-0431-1 (PMC5331692; doi:10.1186/s13045-017-0431-1)
Supplement: Additional file 2: Table S2. — 112 genes covered by a custom targeted NGS gene panel. (DOCX 94 kb) [file 13045_2017_431_MOESM2_ESM.docx]

Additional file 2: Table S2. 112 genes covered by A custom targeted NGS gene panel

| ABL1 | AKT1 | ALAS2 | BIRC3 | XIAP | ATM | CCND1 | BCL2 | BCL6 | PRDM1 |
| --- | --- | --- | --- | --- | --- | --- | --- | --- | --- |
| BRAF | CALR | RUNX1 | CBL | CCND3 | CDKN1A | CEBPA | LYST | CREBBP | CSF3R |
| CUX1 | CYLD | DDX3X | DNM2 | DNMT3A | EGFR | EP300 | EPHA7 | ETV6 | EZH2 |
| FANCA | FANCC | FANCG | FAT1 | FGFR3 | FLT3 | GATA2 | GATA3 | IDH1 | IDH2 |
| IL7R | MUM1 | ITK | JAK1 | JAK2 | JAK3 | KIT | KRAS | SH2D1A | MAF |
| MPL | MYC | MYD88 | MYH11 | NF1 | NOTCH1 | NOTCH2 | NPM1 | NRAS | PAX5 |
| PDGFRB | ABCB1 | PIK3CA | PRF1 | MAPK1 | RELN | PTEN | PTPN11 | RAB27A | RB1 |
| XPO1 | SF1 | CXCR4 | KMT2D | ZRSR2 | SMC1A | ARID1A | STX11 | EED | SMC3 |
| ZMYM3 | MAFB | SH2B3 | SF3A1 | IKZF1 | PRMT5 | ADAMTS13 | U2AF2 | DIS3 | SF3B1 |
| SUZ12 | PRPF40B | SAMHD1 | SETBP1 | TET2 | FAM46C | FBXW7 | CRLF2 | PHF6 | ASXL1 |
| UNC13D | ECT2L | SRSF2 | STXBP2 | TAL1 | TNFAIP3 | TP53 | TRAF3 | U2AF1 | WAS |
| WHSC1 | WT1 |  |  |  |  |  |  |  |  |
