# Supplementary material for: Spectrum of somatic mutations detected by targeted next-generation sequencing and their prognostic significance in adult patients with acute lymphoblastic leukemia
Source: J Hematol Oncol. 2017 Feb 28;10:61. doi: 10.1186/s13045-017-0431-1 (PMC5331692; doi:10.1186/s13045-017-0431-1)
Supplement: Additional file 3: — Supplementary: materials and methods. (DOCX 73 kb) [file 13045_2017_431_MOESM3_ESM.docx]

**Supplementary: Materials and methods**

**Sequencing method and Bioinformatics analysis**

Native genomic DNA was extracted from the bone marrow mononuclear cells of 121 ALL patients and library was constructed as per manufacturer’s protocol. A target-specific next generation sequencing (NGS) approach, which combines multiplex PCR-based target enrichment and library generation with ultra-deep high-throughput parallel sequencing using a Ion Proton platform was uesd. We designed a malignant hematologic disorders specific target panel, covering hotspots or complete coding regions of 112 genes known to be recurrently mutated and/or related to malignant hematologic disorders. Exons of these genes were sequenced on an Ion Torrent semiconductor platform and results were mapped to NCBI hg19 RefSeq with a mean of >97% coverage of the targeted regions at an average depth of 800X. Polymorphisms annotated in dbSNP 135 were excluded. All putative mutations were compared against multiple databases (e.g.1000 genomes, COSMIC, PolyPhen, SIFT).
