# Supplementary material for: Spectrum of somatic mutations detected by targeted next-generation sequencing and their prognostic significance in adult patients with acute lymphoblastic leukemia
Source: J Hematol Oncol. 2017 Feb 28;10:61. doi: 10.1186/s13045-017-0431-1 (PMC5331692; doi:10.1186/s13045-017-0431-1)
Supplement: Additional file 4: — Supplementary: statistical analysis. (DOCX 52 kb) [file 13045_2017_431_MOESM4_ESM.docx]

**Supplementary: Statistical analysis**

All statistical analyses were performed using SPSS 23.0 software. Relapse-free survival (RFS) (defined as the time from achieving CR to relapse) and OS (defined as time from diagnosis to death or lost follow-up) were analyzed by Kaplan-Meier method, Cox proportional hazard regression models were used to assess the prognostic relevance of mutated genes and other established survival indicators, such as sex, age, white blood cell (WBC) count. A P value <0.05 (two-sided) was considered as statistically significant.
