# Supplementary figures and images for: Spectrum of somatic mutations detected by targeted next-generation sequencing and their prognostic significance in adult patients with acute lymphoblastic leukemia
Source: J Hematol Oncol. 2017 Feb 28;10:61. doi: 10.1186/s13045-017-0431-1 (PMC5331692; doi:10.1186/s13045-017-0431-1)

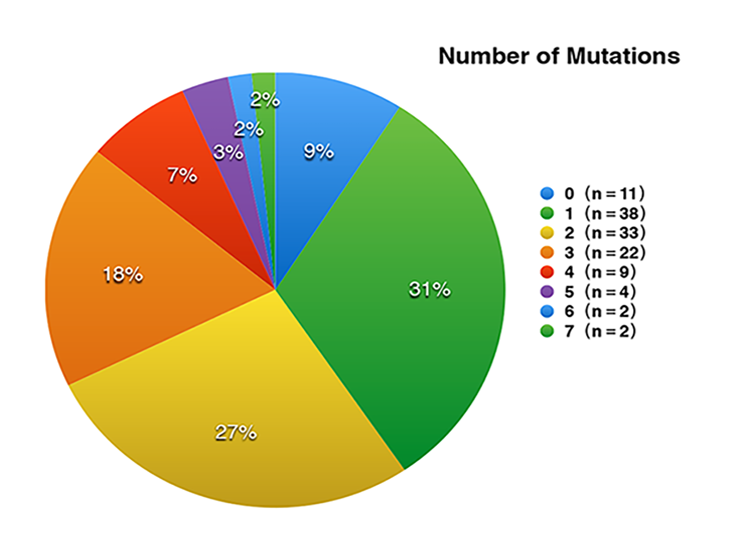

Supplement: Additional file 5: Figure S1. — Percentage of ALL patients with different somatic mutations. (TIF 1433 kb) [file 13045_2017_431_MOESM5_ESM.tif]

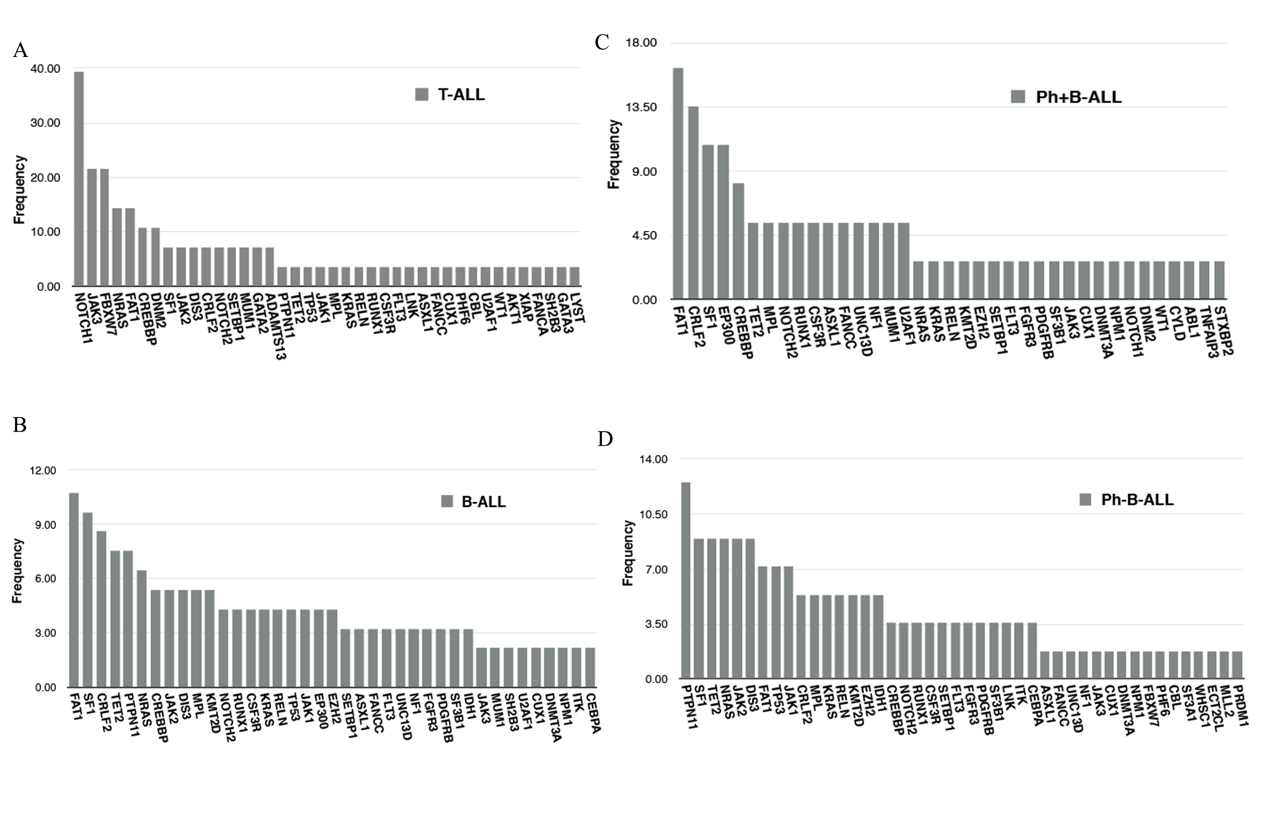


Additional file 6: Fig S2. Frequency of gene mutations in T-ALL (A), in B-ALL (B),in Ph+B-ALL (C), and in Ph-B-ALL (D).

Supplement: Additional file 6: Figure S2. — Frequency of gene mutations in T-ALL (A), in B-ALL (B), in Ph+B-ALL (C), and in Ph-B-ALL (D). (DOCX 4090 kb) [file 13045_2017_431_MOESM6_ESM.docx]

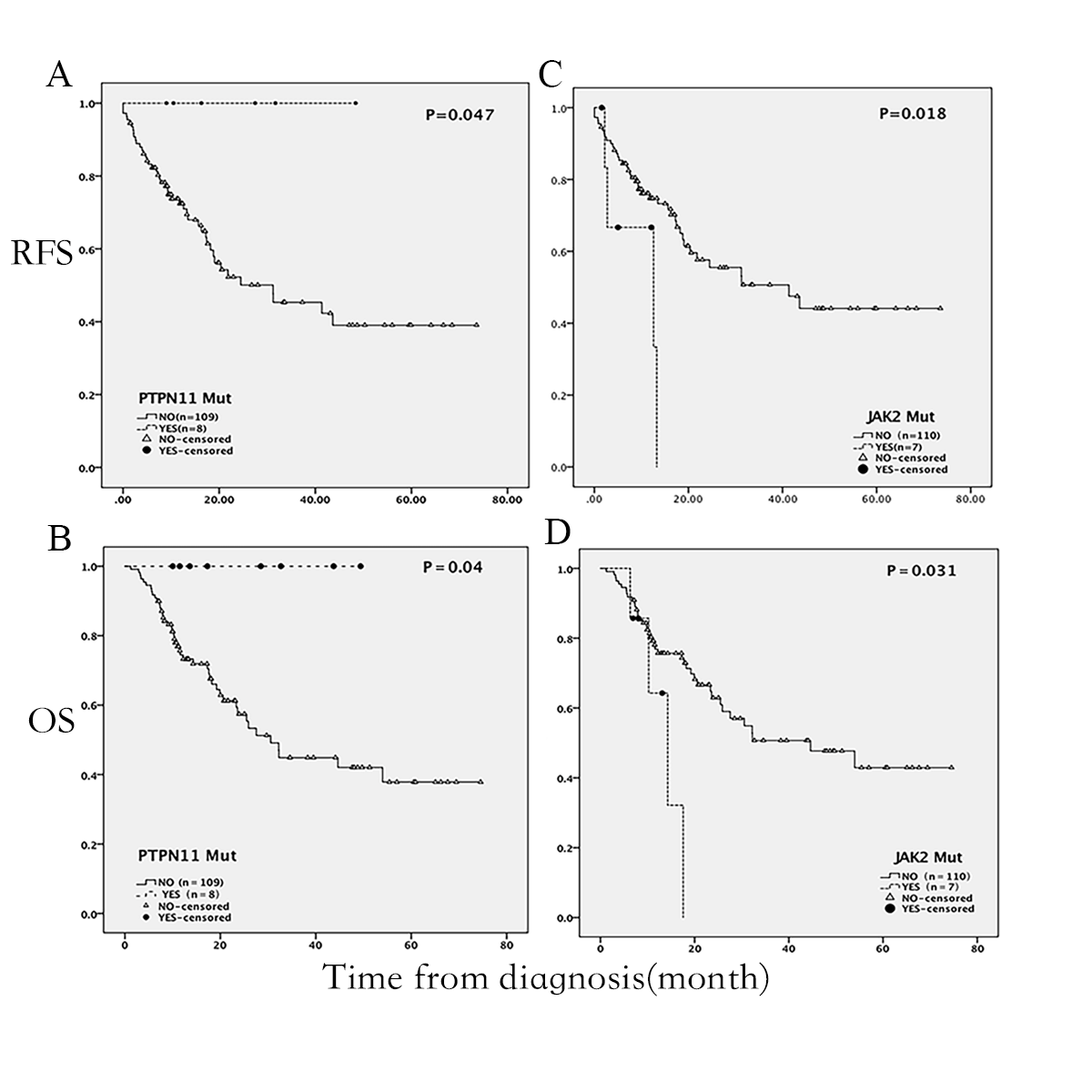

Supplement: Additional file 8: Figure S3. — Probability of RFS or OS for total adult ALL patients with/without PTPN11 and JAK2 mutations. (TIF 4888 kb) [file 13045_2017_431_MOESM8_ESM.tif]

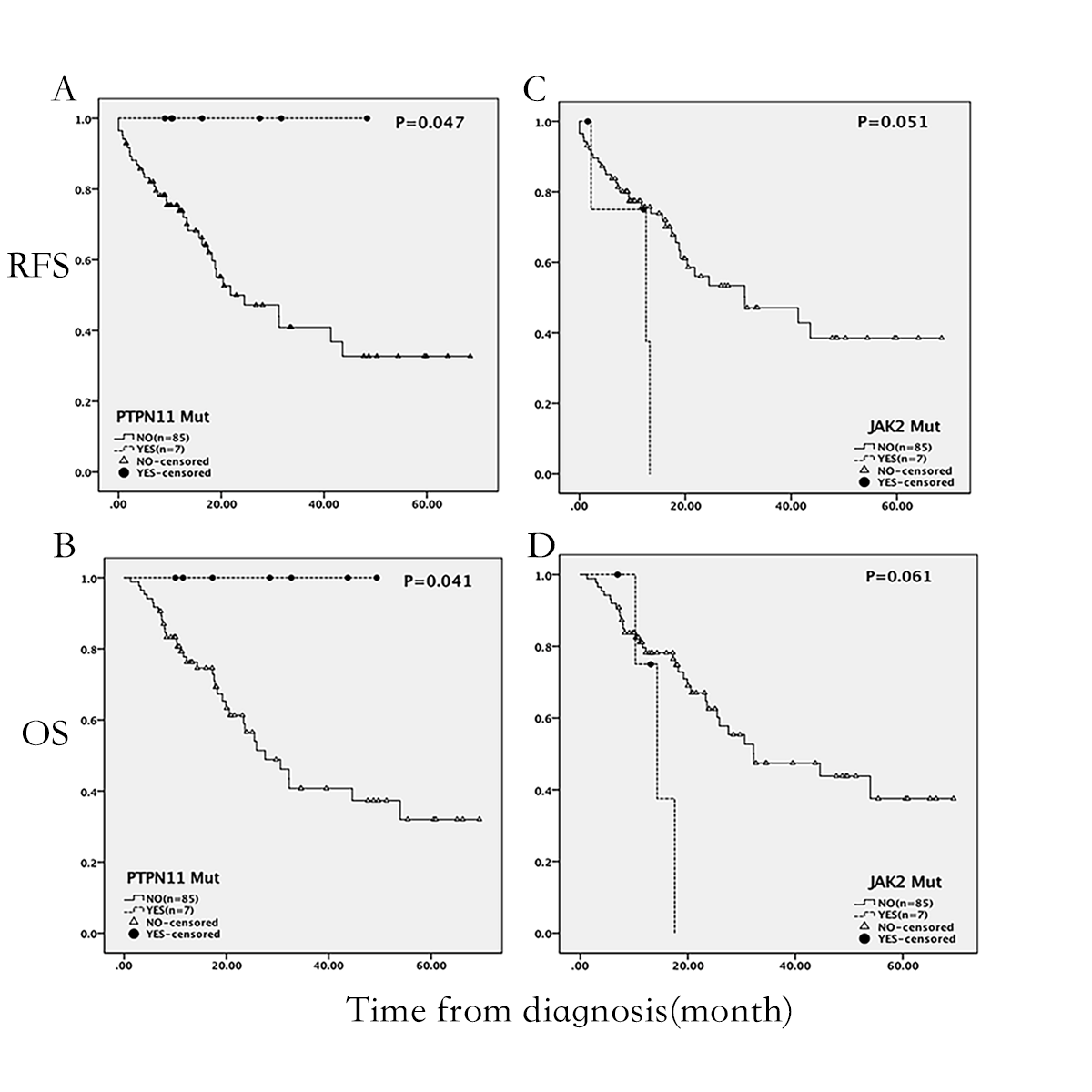

Supplement: Additional file 9: Figure S4. — Probability of RFS or OS in adult B-ALL patients with/without PTPN11 and JAK2 mutations. (TIF 4909 kb) [file 13045_2017_431_MOESM9_ESM.tif]

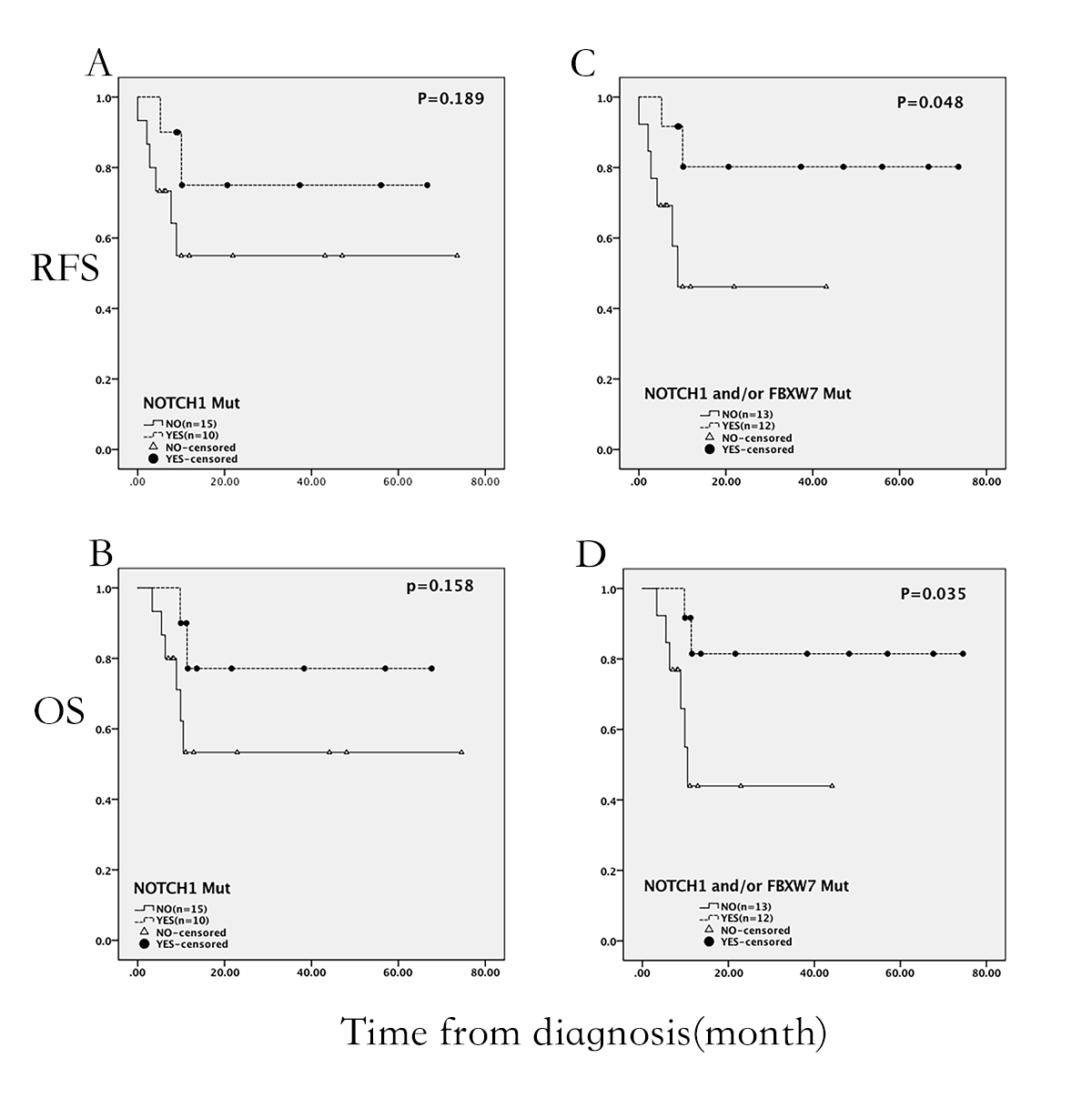

Supplement: Additional file 10: Figure S5. — Probability of RFS or OS in adult T-ALL patients with/without NOTCH1 and/or FBXW7 mutations. (TIF 3370 kb) [file 13045_2017_431_MOESM10_ESM.tif]
