# Supplementary material for: Spectrum of somatic mutations detected by targeted next-generation sequencing and their prognostic significance in adult patients with acute lymphoblastic leukemia
Source: J Hematol Oncol. 2017 Feb 28;10:61. doi: 10.1186/s13045-017-0431-1 (PMC5331692; doi:10.1186/s13045-017-0431-1)
Supplement: Additional file 7: Table S3. — Signal pathways affected and their frequency. (DOCX 70 kb) [file 13045_2017_431_MOESM7_ESM.docx]

Additional file 7：Table S3. Signal pathways affected and their frequency

Pathways Gene Frequency(%)

Transcription CREBBP  RUNX1 29.75

factor/regulation EP300 DNM2

CEBPA PHF6 WT1

PRDM1 GATA2

GATA3 UNC13D ITK

Ras/Protein NRAS KRAS 28.10

phosphatase/MARK PTPN11 NF1 CBL

/PI3K signaling TP53

pathway

epigenetic modulators TNFAIP3 CYLD 28.09

TET2 DNMT3A

IDH1 SETBP1

ASXL1 KMT2D

EZH2 WHSC1

JAK-STAT pathway JAK1 JAK2 JAK3 24.29

CRLF2

NOTCH signaling NOTCH2 NOTCH1 21.49

pathway FBXW7

Splicing and mRNA SF1 U2AF1 DIS3 20.66

processing regulation SF3A1 SF3B1

Receptor/Nonreceptor FGFR3 SH2B3 16.53

tryosine kinase PDGFRB FLT3

signaling pathway ABL1 CSF3R

WNT pathway FAT1 11.57

Other RELN MUM1 10.66

ADAMTS13 LYST

NPM1 CUX1

ECT2L STXBP2

MPL
